# Vitamin A and Histone Deacetylases in Alzheimer’s disease: The Good, the bad and the ugly

**DOI:** 10.1002/alz.093281

**Published:** 2025-01-03

**Authors:** Chhanda Bose, Ashly X Hindle, Adam X Baker, Shane Smith, Jake Strickland, Isabel Guzman, Sharda P Singh, J. Josh Lawrence

**Affiliations:** ^1^ 1Department of Pharmacology and Neuroscience and Garrison Institute on Aging, Lubbock, TX USA; ^2^ Texas Tech University Health Sciences Center, Lubbock, TX USA; ^3^ TTUHSC, Lubbock, TX USA

## Abstract

**Background:**

In Alzheimer’s disease (AD), histone acetylation is disrupted, suggesting loss of transcriptional control. Moreover, converging evidence suggests an age‐ and AD‐dependent loss of transcription controlled by all‐trans‐retinoic acid (ATRA), the bioactive metabolite of vitamin A (VA). Antioxidant depletion causes oxidative stress (OS). Nrf2‐mediated antioxidant defenses are triggered by OS. Here, we investigated roles of VA, histone acetylation, Nrf2, and OS *in vitro*. Finally, we established a dietary dose of vorinostat that crosses the blood brain barrier and promotes histone acetylation in AD mouse brain.

**Method:**

For *in vitro* studies, mouse HT22 cells were treated with vorinostat (up to 40 µM), ATRA, and/or H_2_O_2_. MTT and lipid peroxidation assays were performed. Acetylated histone H3 and Nrf2 expression were examined via western blot (WB) and immunocytochemistry (ICC). For *in vivo* studies, the hAb‐KI AD mice were treated with 0.18 or 0.36 mg vorinostat/gram of diet (125 or 250 mg/kg/week for 2 weeks). HDAC activity in brain tissue was examined via histone H3 acetylation and enzyme activity via WB and colorimetric ELISA.

**Result:**

Vorinostat and ATRA treatment (up to 20 μM) had no significant cytotoxic effects on HT22 cells. H_2_O_2_ alone (25‐50 µM) caused ∼30‐40% cell death (p<0.0001). ATRA (5 µM), in combination with vorinostat (0.5 µM), protected against H_2_O_2_ up to 150 µM. Vorinostat caused gradual and significant (p<0.001) induction in acetylation of histone H3 with 0.5‐3.0 µM treatment for 24h, with no change in total histone H3 levels. ROS was significantly reduced. Nuclear translocation of Nrf2 was induced by H_2_0_2_ and reduced after ATRA and vorinostat treatment. For 0.18 or 0.36 mg vorinostat/gram doses *in vivo*, histone H3 acetylation was increased, HDAC activity was inhibited (p<0.001 and 0.05, respectively), and peroxidation was reduced.

**Conclusion:**

Doses of vorinostat used *in vitro* and *in vivo* increased histone acetylation without cytotoxicity/toxicity. *In vivo*, we established that 125 mg/kg/week in diet is an optimal tolerable dose. VA, alone and in combination with vorinostat, may exhibit synergistic effects in protecting neuronal cells from oxidative stress. Together, our study provides a possible link between oxidative stress, Nrf2 and HDACs as potential contributors to Alzheimer’s disease progression.

